# Systemic inflammation, gut microbiome composition, and cognitive performance among older Ugandans with Alzheimer’s disease and related dementias: A cross-sectional study

**DOI:** 10.1097/MD.0000000000050956

**Published:** 2026-10-02

**Authors:** Kamada Lwere, Noeline Nakasujja, Haruna Muwonge, Hakim Sendagire, Joy Louise Gumikiriza-Onoria, Denis Buwembo, William Buwembo, Samuel Kabwigu, James Kiguli Mukasa, Fred Kirya, Aisha Nazziwa, Emmanuel Othieno, Rita Nassanga, Rheem Nakimbugwe, Ian Guyton Munabi, Martha Sajatovic, Mark Kaddumukasa

**Affiliations:** a Department of Microbiology, School of Biomedical Sciences, College of Health Sciences, Makerere University, Kampala, Uganda; b Department of Microbiology, Faculty of Health Sciences, Soroti University, Arapai, Uganda; c Habib Medical School, Faculty of Health Sciences, Islamic University in Uganda, Mbale, Uganda; d Department of Psychiatry, School of Medicine, College of Health Sciences, Makerere University, Kampala, Uganda; e Department of Physiology, School of Biomedical Sciences, College of Health Sciences, Makerere University, Kampala, Uganda; f School of Public Health, College of Health Sciences, Makerere University, Kampala, Uganda; g Department of Anatomy, School of Biomedical Sciences, College of Health Sciences, Makerere University, Kampala, Uganda; h Department of Obstetrics and Gynaecology, Faculty of Health Sciences, Soroti University, Arapai, Uganda; i Department of Surgery, Faculty of Health Sciences, Soroti University, Arapai, Uganda; j Department of Pathology, Faculty of Health Sciences, Soroti University, Arapai, Uganda; k Department of Radiology, School of Medicine, College of Health Sciences, Makerere University, Kampala, Uganda; l Neurological and Behavioral Outcomes Center, University Hospitals Cleveland Medical Center, Case Western Reserve University School of Medicine, Cleveland, OH; m Department of Medicine, School of Medicine, College of Health Sciences, Makerere University, Kampala, Uganda.

**Keywords:** Alzheimer disease, cognitive performance, C-reactive protein, gut microbiome, systemic inflammation

## Abstract

Systemic inflammation is implicated in neurodegeneration; however, evidence linking circulating inflammatory biomarkers to cognitive decline remains inconsistent, particularly in low- and middle-income countries. High-sensitivity C-reactive protein (hs-CRP) is a standard systemic inflammatory marker; however, its relationship with cognitive performance and gut microbiome composition in sub-Saharan Africa remains underexplored. We investigated the association between hs-CRP and cognitive performance across older Ugandans diagnosed with Alzheimer disease (AD), mild cognitive impairment (MCI), and cognitively intact controls and examined whether gut microbial composition modified this relationship. We conducted a cross-sectional study of community-dwelling older adults in Wakiso District, Uganda. Cognitive performance was assessed using the Education-Adjusted Montreal Cognitive Assessment (MoCA). Systemic inflammation was quantified using plasma hs-CRP, and gut microbial profiles were generated via full-length 16S rRNA gene sequencing. Multivariable linear regression models were used to evaluate the associations between log-transformed hs-CRP concentrations and MoCA scores, adjusting for age, sex, and body mass index. Linear regression assumptions were formally tested. Exploratory analyses examined taxon-level differential abundance across diagnostic categories, hs-CRP–microbiome correlations, and hs-CRP × taxon interaction terms on MoCA scores, with multiple-testing adjustment using the false discovery rate. The analytic cohort comprised 85 participants (mean age 77.5 ± 9.2 years; 67 AD, 10 MCI, and 8 controls). Median hs-CRP was higher in AD (1.96 [0.25–3.75] mg/L) than in MCI (0.25 [0.25–2.73] mg/L) and controls (0.25 [0.25–0.84] mg/L). Higher hs-CRP showed a modest inverse trend with MoCA scores that was not statistically significant after adjustment for age, sex, and body mass index (standardized β=−0.18, P=.11). Advancing age was independently associated with poorer cognitive performance (standardized β=−0.29, P=.013). Several microbial genera exhibited nominal differences across diagnostic groups and hs-CRP levels; however, no individual taxon-level associations or hs-CRP × microbiome interaction terms survived false discovery rate correction (q>0.05). Circulating hs-CRP was not independently associated with global cognitive performance in this cohort of older Ugandans. Genus-level microbiome analyses yielded exploratory, hypothesis-generating patterns that warrant validation in larger longitudinal cohorts investigating gut–brain axis interactions in African populations.

## 1. Introduction

Alzheimer disease (AD) and related dementias constitute an escalating global public health concern, with approximately 55 million individuals currently affected by dementia worldwide, and projections indicating an increase to over 139 million by 2050.^[1,2]^ It is anticipated that approximately two-thirds of this growth will occur in low- and middle-income countries (LMICs), primarily due to population aging.^[3]^ In sub-Saharan Africa, the prevalence of dementia among adults aged ≥ 60 years is estimated to range from 2 to 8%.^[4,5]^ However, these figures likely underestimate the actual burden due to limited diagnostic capabilities, low awareness of cognitive disorders, and restricted access to specialist services. Consequently, dementia is often diagnosed at an advanced stage, which limits the opportunities for early detection. In this context, there is an increasing interest in identifying biological indicators of systemic processes that may facilitate population-level characterization of dementia risk and related pathophysiology, provided that these indicators are interpretable and feasible for implementation in resource-limited settings.

Systemic inflammation has been implicated in neurodegenerative processes through multiple biological pathways.^[6]^ Peripheral immune activation is associated with the release of pro-inflammatory mediators that may influence blood–brain barrier integrity, microglial activation, and oxidative stress within the central nervous system.^[7]^ These processes have been linked to pathological features of AD, including amyloid-β accumulation, tau phosphorylation, and synaptic dysfunction.^[8]^ However, the extent to which circulating inflammatory markers accurately reflect brain-specific inflammatory processes remains unclear, particularly in older populations with substantial comorbidities and age-related immune changes.

C-reactive protein (CRP) is a widely used marker of systemic inflammation and reflects downstream hepatic responses to cytokine signaling, including interleukin-6.^[9–11]^ When measured using high-sensitivity assays (hs-CRP), CRP is commonly interpreted as reflecting low-grade chronic systemic inflammation, with concentrations < 1 mg/L considered low, 1 to 3 mg/L considered intermediate, and > 3 mg/L considered high inflammatory burden. Values ≥ 10 mg/L are generally interpreted as indicative of acute inflammatory states rather than chronic processes. Although CRP is not specific to neuroinflammation, it provides an integrative, albeit nonspecific, indicator of low-grade chronic inflammatory activity and is routinely available in clinical laboratories.^[12,13]^ Its low cost and standardized measurement make CRP a pragmatic candidate for population-based studies of inflammation in LMIC contexts, where access to more specialized biomarkers may be limited.

Evidence from high-income countries suggests that elevated CRP levels are associated with cognitive decline, mild cognitive impairment (MCI), and dementia, although the reported associations are generally modest and inconsistent.^[14–17]^ Several longitudinal studies have indicated that midlife inflammation may be more strongly associated with later cognitive outcomes than inflammation measured in late life, while cross-sectional findings in older adults have been variable and often attenuated after accounting for age and comorbidity.^[18]^ Differences in study design, age at measurement, vascular comorbidity, genetic background, and environmental exposure likely contributed to this heterogeneity.^[19]^ Importantly, data examining the association between systemic inflammation and cognitive performance in African populations, particularly community-based older cohorts, are scarce.

Older adults in sub-Saharan Africa experience distinct life-course exposures, including recurrent infectious diseases, nutritional transitions, and variable access to preventive healthcare, all of which may influence baseline inflammatory profiles.^[20]^ In addition, survival into advanced old age in many sub-Saharan African settings may reflect biological selection processes that further shape the systemic inflammatory profiles in older cohorts. These contextual factors complicate the extrapolation of findings from high-income settings and underscore the need for region-specific evidence. Concurrently, the increasing use of culturally adapted cognitive assessment tools, including education-adjusted versions of the Montreal Cognitive Assessment (MoCA), has improved the identification of cognitive impairment in African populations, facilitating a more reliable investigation of the biological correlates of cognitive aging.^[21]^

Beyond the consideration of systemic inflammation alone, there is an increasing interest in the potential role of the gut microbiome in modulating host immune responses. The composition of the gut microbiota influences inflammatory signaling and metabolic processes, with variations in microbial profiles observed among individuals with neurodegenerative diseases.^[22,23]^ In African populations, where dietary habits and environmental exposures differ significantly from those in Western contexts, the interaction between gut microbial composition and systemic inflammatory markers may occur in ways that are not yet thoroughly characterized.^[23]^ However, empirical data investigating whether features of the gut microbiome may modify associations between CRP and cognitive performance remain limited, particularly in LMIC.^[24]^

Uganda provides a relevant setting to examine these relationships. Previous studies among older Ugandans have characterized cognitive performance using standardized neuropsychological assessments and have identified microbiome-related correlates of AD.^[25]^ Integrating systemic inflammatory measures into this framework allows the evaluation of whether CRP is associated with cognitive performance in this population and whether such associations vary according to gut microbial composition. Clarifying these relationships may inform the interpretation of CRP level as a population-level marker of inflammation in aging African cohorts.

Therefore, the primary aim of this study was to examine the association between systemic inflammation, measured using CRP, and cognitive performance among older Ugandans with AD, MCI, and cognitively healthy controls. The secondary aims were to compare CRP levels across diagnostic groups and explore whether gut microbial composition modifies the relationship between systemic inflammation and cognitive performance. Through these analyses, we sought to provide context-specific evidence on the utility and limitations of CRP as an indicator of systemic inflammation in studies on cognitive aging in sub-Saharan Africa.

## 2. Methods

### 2.1. Study design and participants

This cross-sectional study was conducted between May and July 2023 in Wakiso District, Uganda, which is located in the Greater Kampala Metropolitan Area and includes urban, suburban, and rural areas. The study period was chosen to align with stable community activity levels, ensure participant availability, and minimize seasonal variations in cognitive performance. This research focused on 3 subcounties, Nansana (urban), Busukuma (rural), and Nakwero (rural), which were selected to capture variations in educational attainment and cognitive performance across different socioeconomic backgrounds. These subcounties have distinct educational and occupational profiles, with urban populations generally having greater access to formal education than rural populations. All participants or their legally authorized representatives provided written informed consent.

### 2.2. Study population

The study population comprised community-dwelling adults aged ≥60 years recruited from urban and peri-urban settings in Wakiso District, including Nansana and Busukuma. The participants were drawn from a previously established community-based dementia cohort of older adults who had undergone standardized cognitive screening and clinical characterization. Recruitment for the parent cohort was facilitated by local community leadership structures, including Local Council leaders and Village Health Teams, to ensure broad community representation.

In accordance with the Strengthening the Reporting of Observational Studies in Epidemiology reporting guidelines, participant recruitment and enrollment flows were systematically tracked. From the parent community cohort, 115 eligible older adults were approached and screened. All evaluated individuals completed the full cognitive assessment battery. Thirty individuals were excluded prior to downstream profiling: 12 were excluded due to recent systemic antibiotic exposure within the preceding 6 weeks, 7 were excluded due to active acute illnesses or unstable chronic systemic conditions at the time of sampling, and 11 were excluded due to insufficient biospecimen volume (plasma or rectal swab) for concurrent hs-CRP immunoassay and full-length 16S rRNA gene sequencing. Consequently, a final analytic cohort of 85 participants – comprising 67 individuals with AD, 10 with MCI, and 8 cognitively healthy controls – was included in the present analyses.

### 2.3. Clinical assessment, diagnostic protocols, and consensus adjudication

#### 2.3.1. Cognitive screening and neuropsychological battery

All participants were recruited from a community-based cohort and underwent initial cognitive screening using the Education-Adjusted MoCA and the Identification and Intervention for Dementia in Elderly Africans Screening Instrument. Comprehensive neuropsychological profiling was performed using a harmonized, culturally adapted test battery that evaluated key cognitive domains. Memory and learning were assessed using the Consortium to Establish a Registry for Alzheimer Disease Word List (Immediate and Delayed Recall) and the Craft Story 21 (Immediate and Delayed Recall). Visuospatial and constructional abilities were evaluated using the Stick Design Test and the Benson Complex Figure Copy and Recall tests. Executive function, attention, and processing speed were measured using the Trail Making Tests A and B, Number Span Test (Forward and Backward), and Phonemic Verbal Fluency. Language and semantic retrieval were assessed using the Modified Multilingual Naming Test and Category Verbal Fluency.

#### 2.3.2. Functional, neuropsychiatric, and frailty assessments

Functional independence, informant-reported cognitive decline, and psychiatric symptoms were evaluated to ensure diagnostic precision. Daily functional abilities and dementia severity staging were measured using the Clinical Dementia Rating scale, the Informant Questionnaire on Cognitive Decline in the Elderly, the Identification and Intervention for Dementia in Elderly Africans Instrumental Activities of Daily Living scale, and the National Alzheimer Coordinating Center Functional Assessment Scale. Affective and behavioral profiles were systematically evaluated using the Neuropsychiatric Inventory Questionnaire, the Geriatric Depression Scale, and the Tanner Screening Instrument. Physical frailty and environmental exposures were documented using the Survey of Health, Ageing and Retirement in Europe Frailty Scale, the Social and Environmental Determinants of Health Survey, and the Household Air Pollution Assessment.

#### 2.3.3. Diagnostic classification and consensus adjudication

The final diagnostic classification as AD, MCI, or cognitively normal control was determined through formal multidisciplinary consensus by 2 psychiatrists and a neuropsychologist applying standard Diagnostic and Statistical Manual of Mental DisordersDSM-5 criteria. AD was defined as progressive objective impairment in memory and at least one additional cognitive domain, accompanied by demonstrable impairment in daily functional activities (Clinical Dementia Rating ≥1.0 and Instrumental Activities of Daily Living impairment). MCI was defined as objective impairment in one or more cognitive domains on formal neuropsychological testing with largely preserved basic functional independence (CDR=0.5). Cognitively normal controls were defined as individuals with normative age- and education-adjusted cognitive screening scores, absence of functional decline (CDR=0), and no clinical history of cognitive impairment. Controls were primarily spouses of participants with cognitive impairment to minimize unmeasured confounding from shared environmental, household, and dietary exposures.

#### 2.3.4. Differential diagnosis and exclusion of alternative etiologies

A structured differential diagnostic protocol was applied to systematically rule out non-AD causes of cognitive impairment. Vascular and mixed dementias were excluded through detailed cardiovascular and stroke history, physical neurological examination, and evaluation of vascular risk profiles. Lewy body dementia and frontotemporal dementia were excluded based on the absence of fluctuating cognition, core motor parkinsonism, visual hallucinations, prominent early behavioral changes, or primary progressive language variants. Depression-related pseudodementia and delirium were systematically ruled out using the Geriatric Depression Scale, Neuropsychiatric Inventory Questionnaire affective domains, and clinical evaluations of acute onset or fluctuating consciousness. In addition, individuals with HIV infection, severe systemic illnesses (including chronic kidney disease, acute sepsis, congestive heart failure, or uncontrolled diabetes), active acute illness at the time of sampling, or antibiotic exposure within the preceding 6 weeks were excluded to minimize confounding of inflammatory and gut microbiome measures.

#### 2.3.5. Absence of fluid biomarkers and structural neuroimaging

Because cerebrospinal fluid biomarkers (such as A beta 42 and p-tau), amyloid or tau positron emission tomography, and structural neuroimaging (CT or MRI) were unavailable in this resource-limited, community-based setting, all diagnoses were clinical-syndromic consensus classifications supported by standardized neuropsychological, functional, and psychiatric assessments.

#### 2.3.6. Measurement of systemic inflammation

Systemic inflammation was assessed using plasma CRP, measured in milligrams per liter (mg/L) using a high-sensitivity immunoassay on the Fluorecare analyzer (Fluorecare), with sensitivity appropriate for the detection of low-grade inflammatory variation. The assay lower limit of detection was 0.25 mg/L. CRP concentrations below the detection limit were assigned a value of 0.25 mg/L for analytical purposes. Due to the right-skewed distribution, CRP values were log-transformed for regression analyses and *z*-standardized where appropriate.

For descriptive purposes, hs-CRP concentrations were additionally categorized using a commonly applied interpretive threshold (≥3 mg/L) to indicate higher levels of low-grade systemic inflammation, consistent with prior epidemiological literature. This categorization was used solely for descriptive summaries and supplementary logistic regression analyses and was not intended to define the presence or absence of inflammation.

### 2.4. Gut microbiome profiling and bioinformatic processing

#### 2.4.1. Sample collection and storage

Rectal swabs were collected by a trained laboratory technician at participants’ homes using sterile techniques. The swab tip was gently inserted up to 3 cm into the rectum and rotated for approximately 5 seconds before withdrawal. Swabs were placed immediately into transport tubes and transferred to the laboratory on ice. Upon arrival, the samples were suspended in 2 mL of ASL lysis buffer and stored at − 20°C. DNA extraction was conducted within 2 weeks of collection.

#### 2.4.2. DNA extraction and full-length 16S amplification

Total genomic DNA was isolated from thawed rectal swabs using the modified QIAamp Fast DNA Stool Mini Kit (Qiagen) according to the manufacturer’s protocol. Near full-length 16S rRNA gene sequences (~1465 bp) were amplified using the universal primers 27F (5′-AGAGTTTGATCMTGGCTCAG-3′) and U1492R (5′-TACGGYTACCTTGTTAYGACTT-3′; Inqaba Biotec East Africa Ltd.). PCR amplifications were performed on an Applied Biosystems SimpliAmp Thermocycler (Thermo Scientific) under the following conditions: initial denaturation at 98°C for 5 minutes; 35 cycles of denaturation at 98°C for 30 seconds, annealing at 55°C for 30 seconds, and extension at 72°C for 1 minutes; followed by a final extension at 72°C for 10 minutes. Amplicons were purified using CleanPCR magnetic beads (CLEAN NA) and quantified using a Qubit 4 Fluorometer (Thermo Fisher Scientific).

#### 2.4.3. Library preparation and Oxford Nanopore sequencing

Sequencing libraries were prepared with 20 ng of purified amplicon DNA using the Rapid Barcoding Kit (SQK-RBK114.96; Oxford Nanopore Technologies). Libraries and internal control DNA were loaded onto Flongle Flow Cells (FXP-FSE002) and sequenced on the MinION Mk1B platform (Oxford Nanopore Technologies) over a 24-hours run. Basecalling and quality filtering were executed using MinKNOW/Guppy; reads with a mean quality score (Q-score) <9 or length <200 bp were filtered out. High-quality reads were taxonomically mapped against the SILVA reference database (Release 138) using Minimap2 to generate operational taxonomic units and genus-level abundance matrices.

*Microbiome data filtering, transformation, and modeling*: Taxonomic abundance profiles were processed in R (v4.3.3) using phyloseq and vegan. Genera present in ≥20% of samples were retained for primary multivariable modeling, with a ≥10% threshold evaluated in sensitivity analyses. To address the non-normal, compositional nature of relative abundance data in linear regression models, proportions were transformed using the arcsine square-root transformation and subsequently *z*-standardized. Complementary nonparametric rank tests (Spearman ρ, Kruskal–Wallis) and false discovery rate (FDR) adjustments (Benjamini–Hochberg procedure) were implemented across all diagnostic group comparisons, hs-CRP associations, and hs-CRP × taxon interaction models.

Relative abundance data were transformed using the arcsine square-root transformation to stabilize variance across bounded compositional proportions for exploratory linear modeling. To guard against potential compositional artifacts and test model robustness, all parametric findings were cross-validated using nonparametric rank-based methods (Spearman rank correlations) and prevalence threshold filtering.

### 2.5. Covariates

All multivariate analyses were adjusted for a priori-selected covariates, including age (years, continuous), sex (male/female), and body mass index (BMI, kg/m^2^). The diagnostic group (Alzheimer, MCI, or cognitively healthy control) was additionally included as a covariate in models examining associations across the full sample and omitted in stratified analyses. These variables were selected based on their biological plausibility and prior evidence demonstrating their association with systemic inflammation, gut microbiome composition, and cognitive performance. Covariate selection was intentionally restricted to factors that were measured consistently across all participants to ensure model stability and comparability.

### 2.6. Sample size calculation

Because the analytic sample was constrained by the availability of complete cognitive, hs-CRP, and gut microbiome data within a previously characterized community-based cohort, the statistical power was evaluated using a detectable-effect framework aligned with the primary analytic models.

*Primary pooled model*: For the primary analysis, we estimated the power for a multivariate linear regression examining the association between hs-CRP and global cognitive performance (MoCA) in the full sample, adjusting for age, sex, BMI, and diagnostic group (AD, MCI, and cognitively healthy controls). Power was assessed for the hypothesis test of the hs-CRP coefficient (two-sided α = 0.05), which corresponds to a partial association between hs-CRP and MoCA, conditional on the covariates.

With a total sample size of N = 85 and adjustment for 4 prespecified covariates, the test has degrees of freedom, *f* = N−*m*−2, where *m* = 4 Under standard assumptions for multiple linear regression and modest collinearity, detectable-effect calculations indicated that the study had approximately 80% power to detect a moderate partial association (partial correlation), $r_{p}\approx 0.30$ which corresponds to a standardized regression coefficient (β) of approximately 0.30 to 0.35 when variables are standardized. These effect sizes were consistent with those reported in prior late-life studies on CRP and cognitive performance.

*Group-stratified and interaction models*: Secondary analyses included group-stratified models (e.g., AD only) and CRP × microbiome interaction terms. Given the smaller effective sample sizes within strata and the increased parameterization of the interaction models, these analyses were not powered for definitive hypothesis testing. Accordingly, they were designated as exploratory a priori, with interpretation focused on effect directionality, magnitude, and biological plausibility rather than statistical significance after multiple-testing correction.

### 2.7. Statistical analysis

Participant characteristics were summarized by the diagnostic group using means and standard deviations for approximately normally distributed continuous variables, medians with interquartile ranges for skewed variables, and frequencies with percentages for categorical variables. These summaries describe the demographic, clinical, inflammatory, and cognitive profiles of the study population.

The association between systemic inflammation and cognitive performance was explored visually using scatter plots with fitted linear regression lines. Multivariable linear regression models were then fitted with the MoCA score as the dependent variable and log-transformed hs-CRP concentration as the primary independent variable, adjusted for age, sex, and BMI. Standard linear regression model assumptions were systematically evaluated prior to inference: linearity was checked using component-plus-residual plots; residual normality was assessed via visual inspection of Q–Q plots and the Shapiro–Wilk test; homoscedasticity was verified using Breusch–Pagan tests; and absence of severe multicollinearity was confirmed with variance inflation factors (all VIF<2.0). Influential observations were evaluated using Cook distance. Both unstandardized and standardized regression coefficients with corresponding 95% confidence intervals are reported.

The association between systemic inflammation and cognitive performance was explored visually using scatter plots with fitted linear regression lines. Multivariable linear regression models were fitted with the MoCA score as the dependent variable and log-transformed CRP concentration as the primary independent variable. The models were adjusted a priori for age (years), sex, and BMI. Both unstandardized and standardized regression coefficients have been reported to facilitate the interpretation and comparison of effect sizes.

Differences in gut microbiome composition by diagnostic status were examined using adjusted linear models comparing genus-level relative abundance between participants with AD and cognitively healthy controls. These models included CRP level, age, sex, and BMI as covariates, with the diagnostic group incorporated where applicable. The results were summarized using volcano plots displaying adjusted effect estimates and corresponding *P*-values.

Associations between systemic inflammation and gut microbial composition were evaluated by regressing the genus-level relative abundance on *z*-standardized CRP values using multivariable linear models adjusted for age, sex, BMI, and diagnostic group. Effect sizes and nominal statistical significance were visualized using volcano plots. As a robustness check, nonparametric Spearman rank correlation analyses were performed, and the results were compared across alternative taxon prevalence thresholds.

To assess whether gut microbial composition modified the association between systemic inflammation and cognitive performance, CRP × taxon interaction terms were incorporated into multivariable linear regression models with the MoCA score as the outcome. These models were adjusted for age, sex, BMI, and diagnostic group. Standardized interaction coefficients were extracted and visualized using volcano plots and heatmaps. Given the number of taxa evaluated and the modest sample size, the interaction analyses were exploratory a priori.

Sensitivity analyses were performed to evaluate the stability of the findings. These included comparisons across different taxon prevalence thresholds (≥10% vs ≥20%), an assessment of influential observations using Cook’s distance, and a comparison of parametric regression estimates with nonparametric correlation results. FDR correction was applied where appropriate, and the results were interpreted cautiously with emphasis on effect magnitude and consistency rather than statistical significance alone.

All analyses were performed using R (version 4.3.3) with packages including dplyr, ggplot2, vegan, and related statistical and visualization libraries. A two-sided *P*-value < .05 was considered nominally statistically significant unless otherwise specified.

### 2.8. Efforts to address potential sources of bias

Selection bias was minimized through community-based, door-to-door recruitment conducted by trained village health teams and clinical research staff rather than clinic-based referrals. Cognitively healthy controls were primarily recruited from participants’ spouses to enhance similarity in environmental and dietary exposures. Diagnostic misclassification was reduced using an education-adjusted cognitive battery and consensus Diagnostic and Statistical Manual-5-based diagnoses by psychiatrists and a neuropsychologist, with systematic exclusion of alternative dementia subtypes. Confounding was addressed through a priori adjustment for age, sex, and BMI in multivariable models. Measurement bias was limited through standardized laboratory procedures for hs-CRP and consistent microbiome preprocessing. Analytical bias from multiple testing was mitigated using FDR correction, and exploratory analyses were interpreted cautiously with emphasis on effect magnitude and consistency.

### 2.9. Ethical approval

This study was conducted in accordance with the Declaration of Helsinki and was approved by the Makerere University School of Health Sciences Research and Ethics Committee (MAKSHSREC-2022-395; approved April 11, 2023) and the Uganda National Council for Science and Technology (HS3097ES). Written informed consent was obtained from all participants with decision-making capacity. For participants whose cognitive impairment affected decision-making capacity, written consent was obtained from a legally authorized representative, typically a caregiver or next of kin. Decision-making capacity was assessed by trained clinicians using clinical judgment informed by the cognitive assessment.

## 3. Results

### 3.1. Participant characteristics

A total of 85 participants were included in the analysis, comprising 67 individuals with AD, 10 with MCI, and 8 cognitively healthy controls (Table 1). Participants across groups were older adults, with mean ages of 78.0 ± 9.5 years in the AD group, 79.1 ± 8.9 years in the MCI group, and 71.6 ± 4.7 years among controls. Females constituted the majority of the participants in all diagnostic categories, representing 82.1% of the AD group, 90.0% of the MCI group, and 87.5% of the control group.

**Table 1:** Participant characteristics.

Variable	AD (n = 67)	MCI (n = 10)	Control (n = 8)
Age, yrs	78.0 ± 9.5	79.1 ± 8.9	71.6 ± 4.7
Female, n (%)	55 (82.1%)	9 (90.0%)	7 (87.5%)
BMI, kg/m^2^	30.3 ± 9.4	25.4 ± 7.0	32.5 ± 8.7
MoCA score	10 [6–14]	14 [13–16]	22 [21–23]
CRP, mg/L	1.96 [0.25–3.75]	0.25 [0.25–2.73]	0.25 [0.25–0.84]

This table summarizes the demographic, anthropometric, cognitive, and inflammatory characteristics of the study participants stratified by the diagnostic group. Values are presented as mean ± standard deviation for age and body mass index (BMI), number (percentage) for sex, median [interquartile range] for Montreal Cognitive Assessment (MoCA) scores, and C-reactive protein (CRP) concentrations. Elevated systemic inflammation was defined as a CRP level ≥ 3 mg/L.

Mean BMI varied across groups, with higher BMI observed among controls (32.5 ± 8.7 kg/m^2^) compared with the AD (30.3 ± 9.4 kg/m^2^) and MCI (25.4 ± 7.0 kg/m^2^) groups. As expected, cognitive performance differed by diagnostic category; median MoCA scores were the lowest among participants with AD (10 [6–14]), intermediate in the MCI group (14 [13–16]), and highest among controls (22 [21–23]).

Median hs-CRP concentrations were higher in the AD group (1.96 [0.25–3.75] mg/L) than in both the MCI (0.25 [0.25–2.73] mg/L) and control groups (0.25 [0.25–0.84] mg/L). Hs-CRP concentrations, ≥3 mg/L indicative of elevated low-grade systemic inflammation, were observed in a subset of participants within the AD group (as reflected by the 75th percentile of 3.75 mg/L), while median levels across all groups remained within the low-to-intermediate range. No participants exhibited acute inflammatory levels ≥10 mg/L.

### 3.2. Systemic inflammation and cognitive performance

In multivariable linear regression analyses adjusted for age, sex, and BMI, higher log-transformed CRP concentrations were associated with lower MoCA scores, although this association was not statistically significant (standardized β = −0.18, standard error [SE] = 0.11, *P* = .11; Table 2). Increasing age was independently associated with lower cognitive performance (standardized β = −0.29, SE = 0.11, *P* = .013).

**Table 2:** Multivariable association between systemic inflammation and cognitive performance.

Predictor	Std_Beta	SE	*t*_value	*P*_value
(Intercept)	−0.017738953	0.11598	−0.152948834	.878833605
scale(log_crp)	−0.175259017	0.107906	−1.624187607	.108370354
scale(age)	−0.28583887	0.112333	−2.544578432	.012915407
genderMale	0.158188982	0.286857	0.551455094	.582898113
scale(bmi)	0.092792874	0.113557	0.817146758	.416333518

This table presents the results of a multivariable linear regression model examining the association between systemic inflammation and cognitive performance, measured using the Montreal Cognitive Assessment (MoCA) scores. The primary exposure was the log-transformed C-reactive protein (CRP) level, modeled as a standardized variable. Covariates included age, sex, and body mass index (BMI). Standardized beta coefficients (Std_Beta), standard errors (SE), *t*-statistics, and *P*-values are reported.

Sex and BMI were not significantly associated with MoCA scores in the adjusted model. Together, these findings indicate that while a higher systemic inflammatory burden, as indexed by CRP, tended to correlate with poorer cognitive performance, age remained the dominant predictor of MoCA score in this cohort.

### 3.3. Unstandardized effect estimates

Unstandardized regression estimates yielded results consistent with the standardized model (Table 3). Higher log-transformed CRP levels were associated with lower MoCA scores, corresponding to a decrease of approximately 0.66 points in the MoCA per unit increase in log-CRP; however, this association was not statistically significant (β = −0.66, SE = 0.41, *P* = .11). Increasing age remained independently associated with lower cognitive performance, with each additional year associated with a 0.18-point reduction in MoCA score (β = −0.18, SE = 0.07, *P* = .013).

**Table 3:** Unstandardized multivariable linear regression model for MoCA scores.

Predictor	Beta	SE	*t*_value	*P*_value
(Intercept)	23.46036503	6.422771897	3.652685384	.000468064
log_crp	−0.661216572	0.407106032	−1.624187607	.108370354
age	−0.177357804	0.06970027	−2.544578432	.012915407
genderMale	0.907783308	1.646159982	0.551455094	.582898113
bmi	0.057946552	0.070913274	0.817146758	.416333518

This table presents unstandardized coefficients from a multivariable linear regression model examining the association between systemic inflammation and cognitive performance measured using the Montreal Cognitive Assessment (MoCA) scores. The primary exposure was log-transformed C-reactive protein (CRP) levels. Covariates included age, sex, and body mass index (BMI). Unstandardized regression coefficients (β), standard errors (SE), *t*-statistics, and *P*-values were reported, allowing direct interpretation of effect sizes on the MoCA scale.

Sex and BMI were not significantly associated with MoCA scores in the adjusted model. Overall, the unstandardized estimates corroborated the standardized findings, indicating a modest but nonsignificant inverse association between systemic inflammatory burden and cognitive performance after accounting for demographic and anthropometric factors.

### 3.4. Factors associated with elevated CRP

In the multivariable logistic regression analysis (Table 4), higher log-transformed CRP levels showed a trend toward increased odds of elevated inflammation (odds ratio = 1.45, 95% confidence interval: 0.97–2.16), although this association was not statistically significant (*P* = .071). Age, sex, and BMI were not significantly associated with elevated CRP levels in the adjusted model.

**Table 4:** Multivariable logistic regression analysis of elevated CRP (≥ 3 mg/L).

Term	OR	LCL	UCL	*P*_value
(Intercept)	0.037043	8.67E-05	15.83463149	.286297
log_crp	1.44587	0.968972	2.157481564	.070979
age	1.047354	0.979816	1.119546905	.173701
genderMale	1.145612	0.213831	6.137684309	.873879
bmi	1.036422	0.967313	1.110469835	.309596

This table presents odds ratios (ORs) from a multivariable logistic regression model evaluating factors associated with elevated systemic inflammation, defined as C-reactive protein (CRP) ≥ 3 mg/L. Predictor variables included log-transformed CRP, age, sex, and body mass index (BMI). Results are reported as odds ratios with 95% confidence intervals (CI) and corresponding *P*-values.

### 3.5. hs-CRP and cognitive performance

The unadjusted relationship between systemic inflammation and cognitive performance is shown in Figure 1. Higher hs-CRP concentrations were associated with lower MoCA scores, with a modest negative trend observed across the hs-CRP range. However, substantial variability in MoCA scores was evident at all hs-CRP levels.

**Figure 1.**
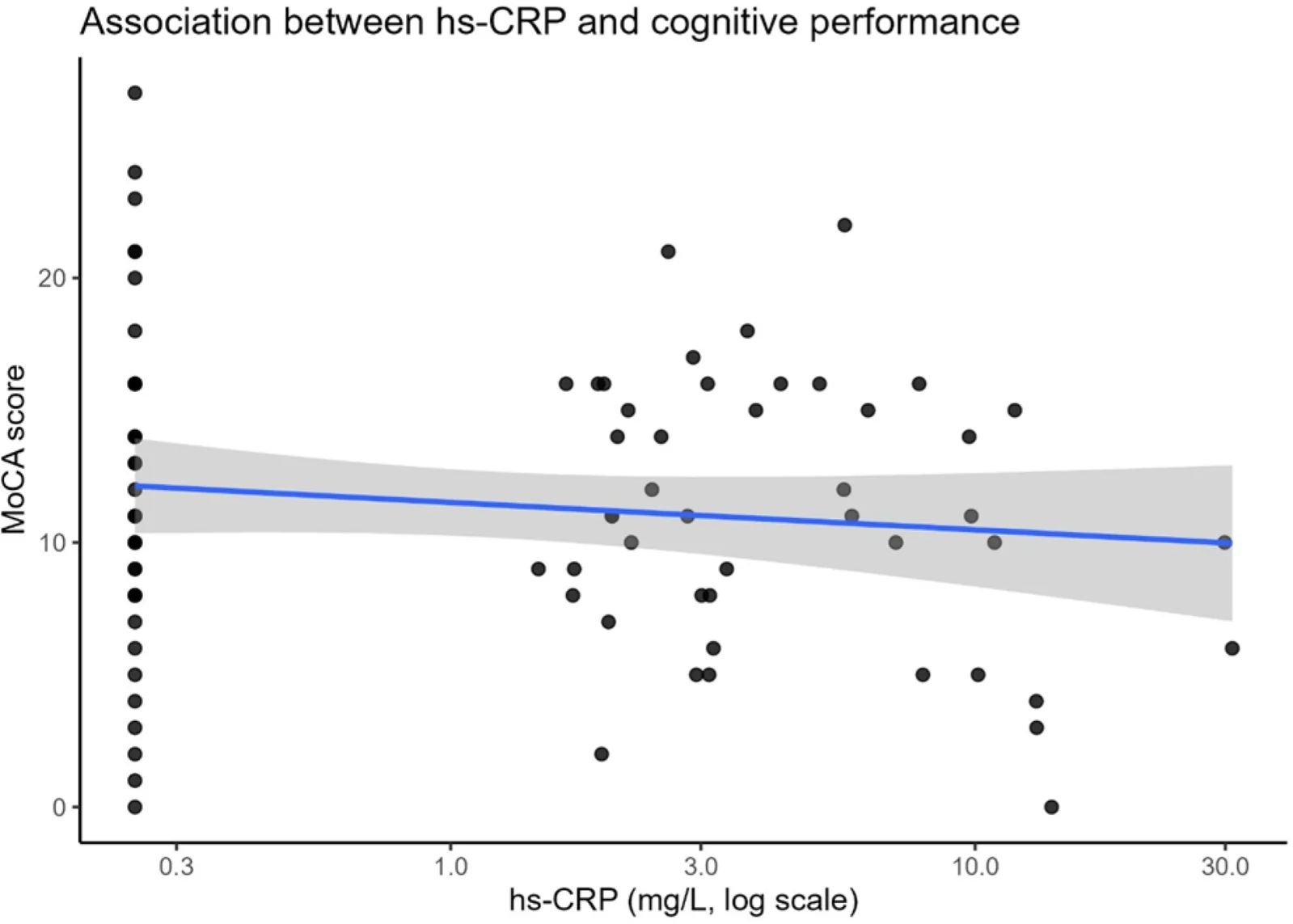
Scatter plot showing the relationship between high-sensitivity C-reactive protein (hs-CRP) concentrations and cognitive performance as measured by the Montreal Cognitive Assessment (MoCA). hs-CRP values are displayed on a logarithmic scale. The solid blue line represents the fitted linear regression, with the shaded area indicating the 95% confidence interval.

Consistent with this visual pattern, multivariable linear regression analyses adjusted for age, sex, and BMI showed that higher hs-CRP levels were associated with lower MoCA scores; however, the association was not statistically significant (β = −0.66, *P* = .108). In contrast, increasing age was independently associated with lower cognitive performance, while sex and BMI were not significantly related to MoCA scores.

### 3.6. Differential gut microbiome composition by diagnostic status

Adjusted comparisons of genus-level relative abundance between individuals with AD and cognitively healthy controls are summarized in Figure 2. After adjustment for hs-CRP, age, sex, BMI, and diagnostic group, several microbial genera demonstrated nominal differences in relative abundance between groups. Genera with negative adjusted mean differences, indicating higher abundance among cognitively healthy controls, included *Ochrobactrum* and members of the *Allorhizobium–Neorhizobium–Pararhizobium–Rhizobium* complex, which showed the largest effect sizes and strongest nominal statistical signals. In contrast, a smaller number of genera, including *Dickeya*, exhibited higher relative abundance in the AD group, although the magnitude of these differences was modest.

**Figure 2.**
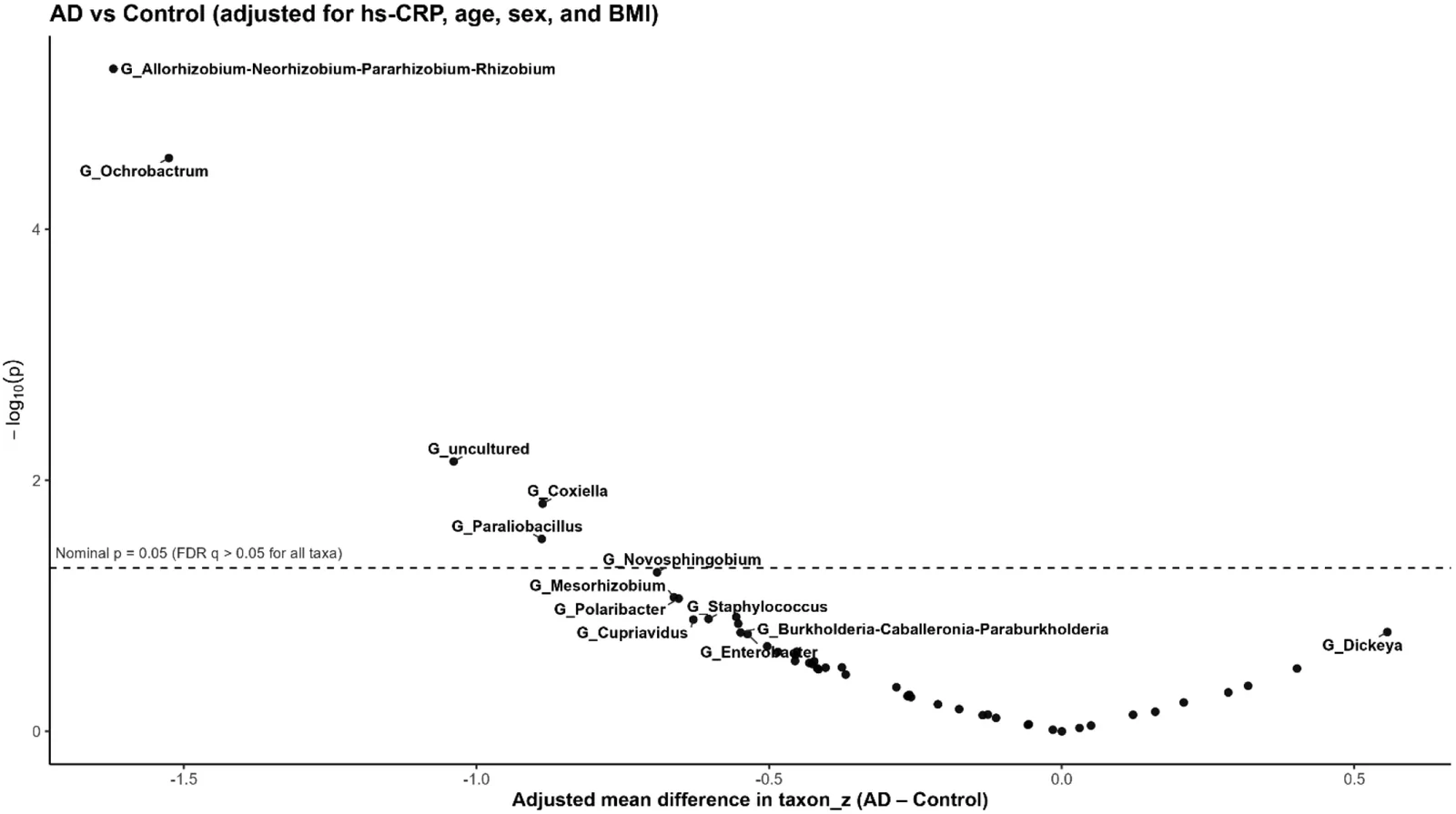
Volcano plot showing adjusted differences in genus-level gut microbial relative abundance between individuals with Alzheimer disease (AD, n=67) and cognitively normal controls (n=8). The horizontal *x*-axis represents the adjusted mean difference in *z*-standardized taxon abundance (AD – Control), where negative values indicate higher relative abundance in controls and positive values indicate higher relative abundance in AD. The vertical *y*-axis represents the $-log_{10}$ nominal *P*-value. Multivariable linear models were adjusted for plasma hs-CRP, age, sex, and body mass index (BMI). The horizontal dashed line marks the nominal significance threshold (P=.05). No bacterial genera remained statistically significant following false discovery rate (FDR) correction (q>0.05). All labeled taxa (e.g., *Allorhizobium-Neorhizobium-Pararhizobium-Rhizobium*, *Ochrobactrum*, and *Dickeya*) represent exploratory, hypothesis-generating findings. hs-CRP = high-sensitivity C-reactive protein.

Most genera clustered near the null, reflecting small adjusted mean differences and limited statistical separation between groups. While several taxa crossed the nominal significance threshold (*P* < .05), none of the observed associations remained statistically significant after correction for multiple testing.

### 3.7. Associations between systemic inflammation and gut microbial taxa

Adjusted linear regression analyses identified several gut microbial genera associated with systemic inflammation, as measured by hs-CRP levels (Fig. 3). Both positive and negative associations were observed, indicating that a higher inflammatory burden is linked to the differential abundance of specific taxa. Genera such as *UCG-002*, *Companilactobacillus*, and *Coxiella* showed positive associations with hs-CRP, whereas *Serratia*, *Vibrio*, *Escherichia–Shigella*, and *Hafnia–Obesumbacterium* were inversely associated.

**Figure 3.**
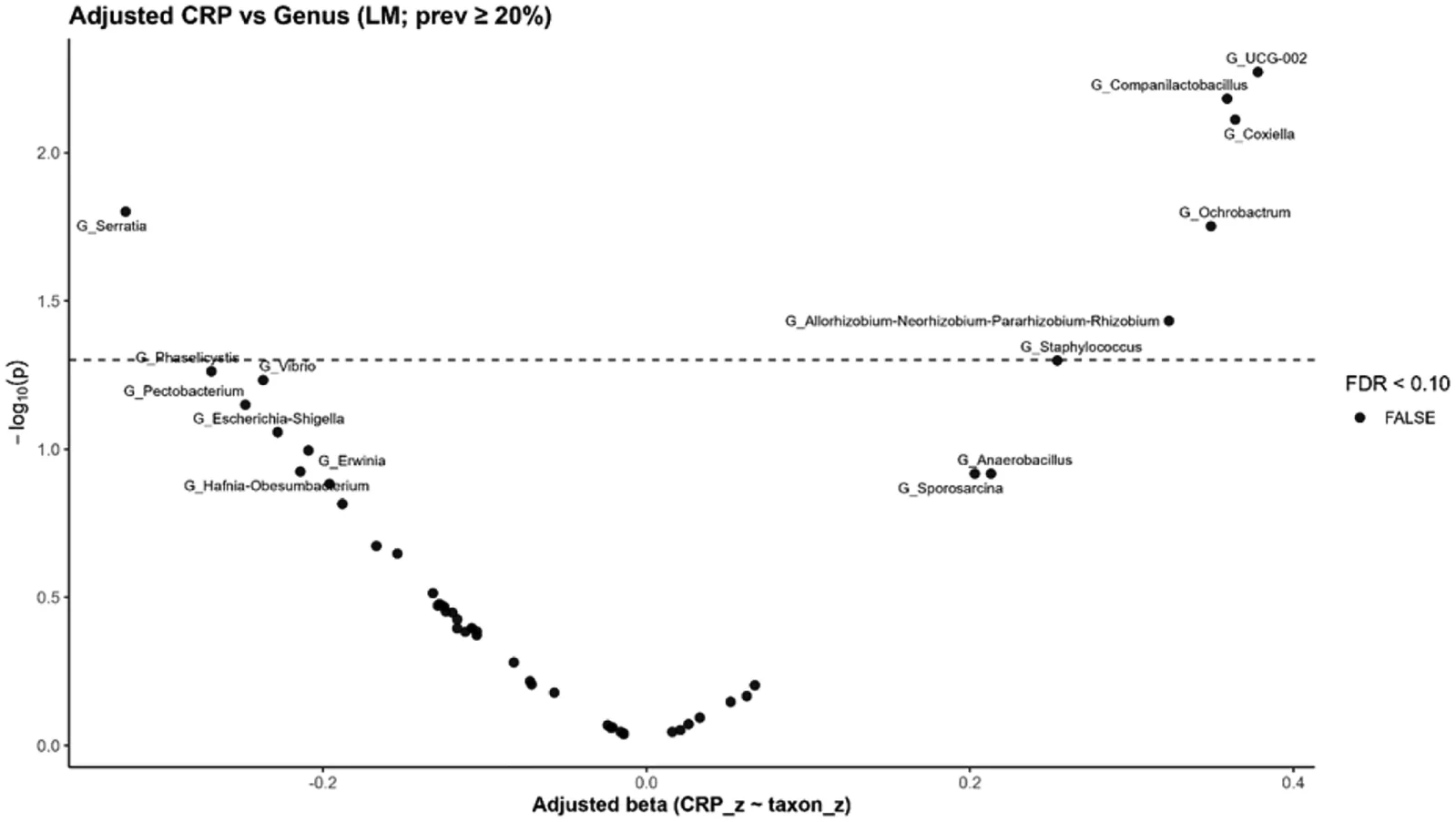
Volcano plot showing associations between high-sensitivity C-reactive protein (hs-CRP) and genus-level gut microbial relative abundance from multivariable linear models. Analyses were restricted to bacterial genera present in at least 20% of samples (prevalence≥20%). The horizontal *x*-axis represents the standardized regression coefficient (β) for the association between *z*-standardized hs-CRP and *z*-standardized taxon abundance. The vertical *y*-axis represents $-log_{10}$ nominal *P*-values. Models were adjusted for age, sex, body mass index (BMI), and diagnostic group. The horizontal dashed line denotes the nominal significance threshold (P=.05). No microbial genera met the false discovery rate threshold (all FDR q≥0.10, indicated as FALSE). All nominally associated taxa (e.g., *UCG-002*, *Companilactobacillus*, *Coxiella*, and *Serratia*) represent exploratory signals requiring cautious interpretation.

Most associations were modest in effect size and did not remain significant after correction for multiple testing, reflecting inter-individual variability and the exploratory nature of genus-level analyses.

### 3.8. Modification of inflammation–cognition associations by gut microbial taxa

Interaction analyses revealed that the relationship between systemic inflammation and cognitive performance varied according to the abundance of specific gut microbial genera (Fig. 4). Several taxa demonstrated nominally significant CRP × genus interaction effects, suggesting that the association between hs-CRP and MoCA scores differed depending on microbial composition.

**Figure 4.**
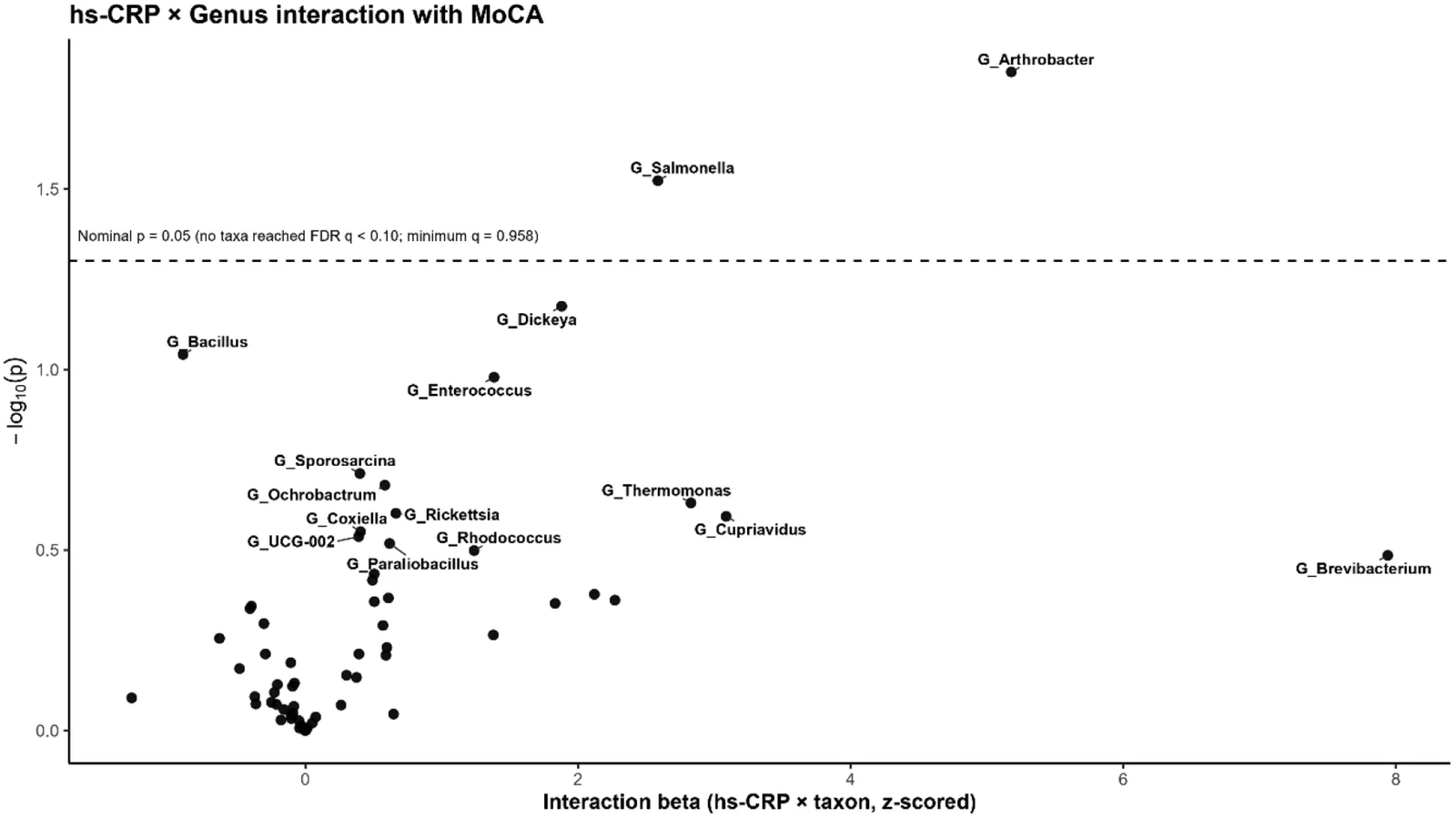
Volcano plot showing exploratory interaction effects between high-sensitivity C-reactive protein (hs-CRP) and genus-level gut microbial abundance on cognitive performance (MoCA scores). The horizontal *x*-axis represents the standardized interaction coefficient ($\beta_{interaction}$) for the hs-CRP×taxon interaction term. The vertical *y*-axis represents $-log_{10}$ nominal *P*-values. Multivariable linear models were adjusted for age, sex, body mass index (BMI), and diagnostic group. The horizontal dashed line denotes the nominal significance threshold (P=.05). No interaction terms met the false discovery rate threshold (all FDR q≥0.10, indicated as FALSE). Only *Arthrobacter* and *Salmonella* demonstrated nominal interaction effects (P=.05), representing exploratory, hypothesis-generating findings in this cross-sectional cohort.

Notably, genera including *Arthrobacter*, *Salmonella*, and *Dickeya* showed relatively larger interaction coefficients, indicating greater divergence in cognitive performance across levels of systemic inflammation at higher taxon abundance. Other taxa, such as *Bacillus*, *Enterococcus*, and *Ochrobactrum,* exhibited modest interaction effects. However, none of the observed interactions remained statistically significant after correction for multiple tests.

### 3.9. Ranking of microbial taxa modifying inflammation–cognition relationships

Figure 5 summarizes and ranks gut microbial genera according to the magnitude of their interaction with systemic inflammation in relation to cognitive performance. Among the taxa examined, *Arthrobacter*, *Salmonella*, *Dickeya*, and *Brevibacterium* exhibited the largest positive interaction coefficients, indicating a greater divergence in MoCA scores across hs-CRP levels at higher relative abundances of these genera.

**Figure 5.**
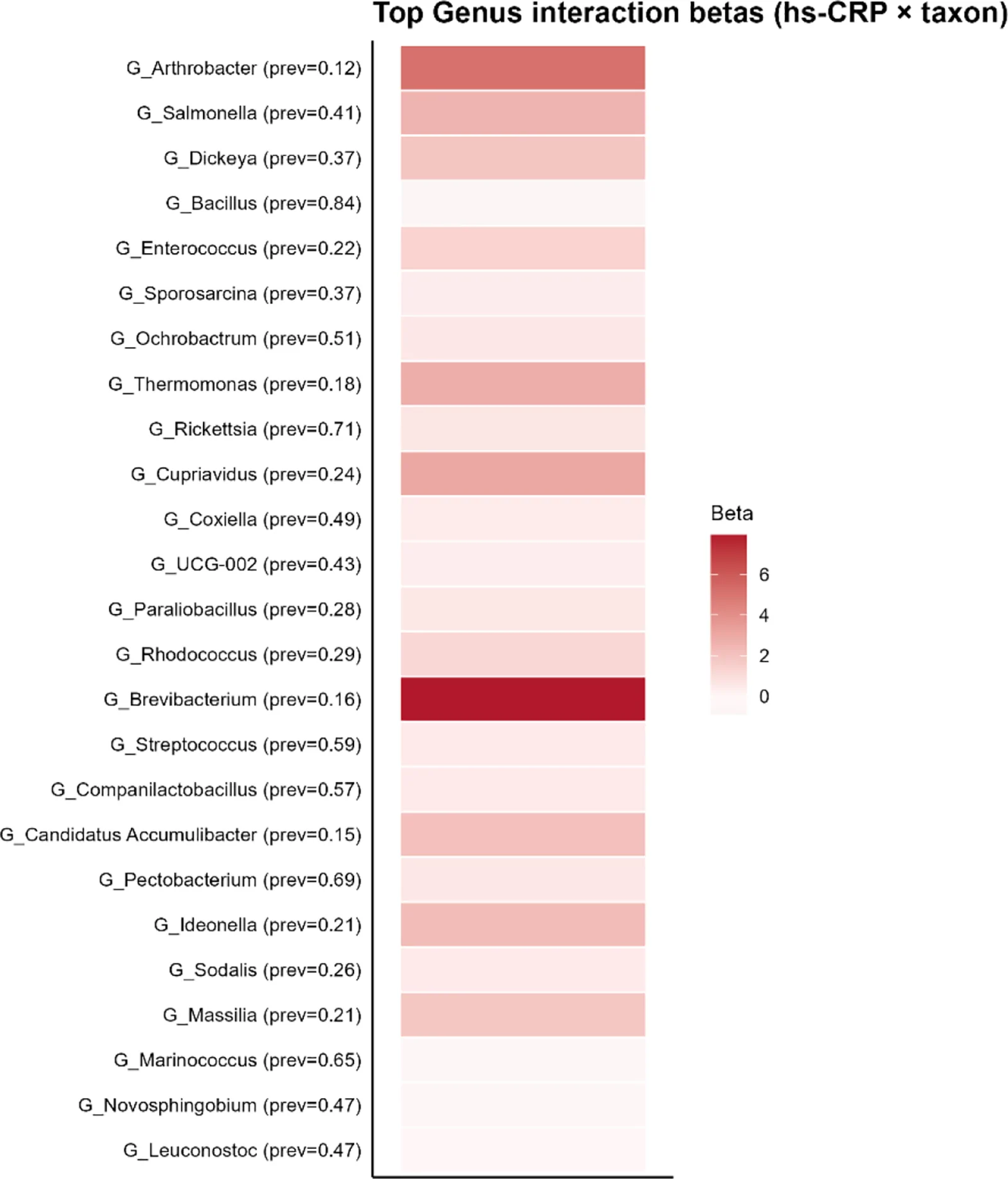
Heatmap displaying standardized interaction coefficients (β) for exploratory high-sensitivity C-reactive protein (hs-CRP) × genus abundance interactions on cognitive performance (MoCA scores). Genera are displayed for taxa with sample prevalence ≥10% (indicated in parentheses). Color intensity represents the magnitude and direction of the standardized interaction beta ($\beta_{interaction}$), with darker red indicating larger positive interaction estimates and light blue indicating negative estimates. Models were adjusted for age, sex, body mass index (BMI), and diagnostic group. No interaction terms remained statistically significant following false discovery rate (FDR) correction (q>0.05). The large interaction estimate observed for low-prevalence taxa (e.g., *Brevibacterium*, prevalence = 0.16) reflects model sensitivity to data sparsity and should be interpreted strictly as an exploratory, hypothesis-generating signal.

Other taxa, including *Bacillus*, *Enterococcus*, *Ochrobactrum*, and *Cupriavidus,* demonstrated moderate interaction effects. Several genera with high prevalence, such as *Marinococcus*, *Novosphingobium*, and *Leuconostoc*, showed minimal interaction with hs-CRP despite being commonly detected.

Consistent with the volcano plot results (Fig. 4), these interaction effects were not statistically significant after correcting for multiple tests. Accordingly, Figure 5 highlights candidate taxa that may warrant further investigation rather than establishing a definitive effect modification.

### 3.10. Cross-method consistency of inflammation–microbiome associations

Figure 6 integrates the results across interaction, adjusted regression, and nonparametric correlation analyses to assess the consistency of inflammation–microbiome associations. Several genera demonstrated concordant directionality across multiple analytical frameworks. For example, *Salmonella*, *Dickeya*, and *Bacillus* showed positive effects in interaction models and corresponding associations in adjusted or correlation analyses, whereas *Serratia* and *Lactococcus* showed inverse relationships with hs-CRP levels.

**Figure 6.**
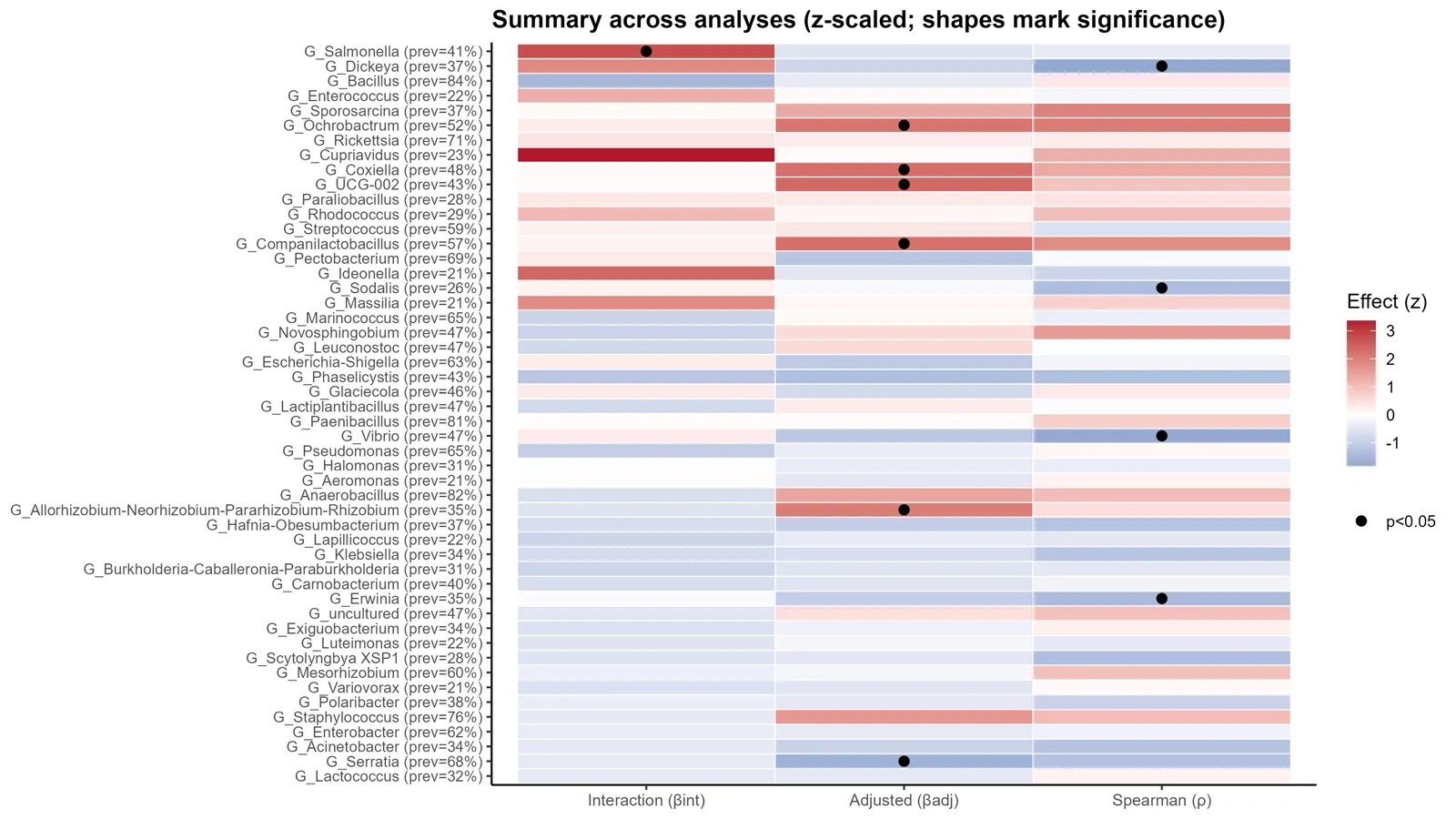
Summary heatmap comparing standardized effect sizes across 3 analytic frameworks for genus-level gut microbial taxa. Standardized effect estimates (z-scaled) are shown for candidate bacterial genera (with sample prevalence indicated in parentheses) across 3 complementary frameworks: multivariable interaction models evaluating high-sensitivity C-reactive protein (hs-CRP) × genus relative abundance interaction terms on Montreal Cognitive Assessment (MoCA) scores adjusted for age, sex, body mass index (BMI), and diagnostic group ($Interaction,\;\beta_{int}$); multivariable linear regression models evaluating associations between plasma hs-CRP and genus relative abundance adjusted for age, sex, BMI, and diagnostic group ($Adjusted,\;\beta_{adj}$); and bivariate nonparametric Spearman rank correlations between genus relative abundance and plasma hs-CRP (Spearman, ρ). Warmer red hues indicate positive associations or interaction effects, whereas cooler blue hues denote inverse associations or interactions. Black circles mark nominal statistical significance (P=.05). Crucially, no associations or interaction terms survived False Discovery Rate (FDR) correction (q>0.05) across any of the 3 frameworks. Discrepancies in effect directionality across analytic frameworks illustrate the sensitivity of compositional microbiome data to modeling assumptions and reinforce the exploratory, hypothesis-generating nature of these findings.

In contrast, other genera exhibited framework-specific associations, with effect directions differing between parametric and nonparametric approaches or between main-effect and interaction models. This heterogeneity highlights the sensitivity of genus-level findings to modeling assumptions and underscores the complexity of host–microbiome–inflammation relationships.

## 4. Discussion

We found that systemic inflammation, measured using hs-CRP, was modestly but not independently associated with global cognitive performance among older Ugandans with AD, MCI, and cognitively healthy controls. Notably, none of the participants exhibited hs-CRP concentrations indicative of high-grade systemic inflammation (≥3 mg/L); however, values within the low-grade chronic inflammation range were observed across all diagnostic groups. This restricted range of hs-CRP variability likely constrains the ability to detect strong independent associations with cognitive performance. In the context of an older cohort, such attenuation may reflect age-related immune regulation and survival effects, as well as long-term adaptation to chronic infectious and environmental exposures common in sub-Saharan Africa. We further observed that several gut microbial genera differed according to diagnostic status and were associated with hs-CRP levels, even after accounting for demographic and clinical factors.

The relatively advanced age of the participants in this study merits consideration. The mean age of individuals with AD and MCI was in the late seventies, which is older than that reported in some clinic-based cohorts from high-income countries. This age distribution likely reflects contextual factors specific to sub-Saharan Africa, including delayed recognition of cognitive impairment, limited access to specialist diagnostic services, and reliance on community-based pathways for case identification. In such settings, individuals with dementia are often identified at advanced ages and stages of the disease. Advanced age may also influence systemic inflammatory profiles, as older individuals often exhibit altered immune regulation and reduced variability in circulating inflammatory markers.

In addition, community-based recruitment may preferentially capture individuals who have survived to an older age despite cumulative life-course exposures, introducing a degree of survivor bias. This may partially explain the restricted range of hs-CRP concentrations within the low-grade chronic inflammation spectrum observed in the cohort and the attenuation of the associations between systemic inflammation and cognitive performance. Previous studies have similarly suggested that the contribution of circulating inflammatory markers to cognitive variance may diminish with advanced age, as age-related neurodegeneration becomes the dominant driver of cognitive decline.

Accordingly, the older age profile of this sample should be viewed as reflective of real-world dementia presentations in Uganda rather than as a limitation of the study design. These findings underscore the importance of considering the age structure and diagnostic context when interpreting inflammation–cognition relationships in LMICs.

### 4.1. Systemic inflammation and cognitive performance

The weak inverse association between hs-CRP and MoCA performance observed in this study is broadly consistent with findings from several cohort and cross-sectional studies conducted in high-income countries, which have reported heterogeneous and often modest relationships between circulating CRP and cognitive outcomes.^[17,26]^ While some longitudinal studies have demonstrated that elevated CRP levels in midlife predict later cognitive decline or dementia, particularly vascular dementia, other investigations in older populations have found null or attenuated associations after accounting for age and comorbidities.^[14,27]^

Fewer studies from sub-Saharan Africa and other LMICs have examined the associations between systemic inflammation and cognitive performance; however, population-based studies have demonstrated substantial heterogeneity in CRP distributions, shaped by age structure, infection burden, and life-course exposures.^[28]^ In many LMIC settings, elevated CRP is more commonly observed in younger or middle-aged adults, likely reflecting cumulative infectious exposure and cardiometabolic transition, whereas cohorts enriched for older adults often exhibit restricted variability in circulating CRP, consistent with survival selection and age-related attenuation of systemic inflammatory responses.^[29]^ Given that our cohort was older than many previously studied LMIC populations, this age group may partly account for the restricted range of hs-CRP concentrations within the low-grade chronic inflammation spectrum observed in this study. Our findings align more closely with the latter body of literature, suggesting that CRP may have limited discriminatory value as a standalone marker of cognitive performance in late life.

In our cohort, age emerged as the strongest independent predictor of lower MoCA scores, whereas hs-CRP levels, sex, and BMI did not show independent associations. This pattern mirrors observations from studies in Europe and North America, showing that the contribution of systemic inflammatory markers to cognitive variance diminishes with advancing age, potentially reflecting cumulative exposure rather than active neurodegenerative processes.^[30,31]^

### 4.2. Gut microbiome differences independent of systemic inflammation

Despite the weak direct association between hs-CRP and cognition, we identified differential abundances of several gut microbial genera between individuals with AD and cognitively healthy controls after adjusting for hs-CRP and demographic factors. This finding is consistent with a growing body of literature demonstrating gut microbiome alterations in AD that are not fully explained by variations in circulating inflammatory markers alone. Studies from high-income settings have similarly reported disease-associated microbial signatures that persist after accounting for systemic inflammation, implicating additional mechanisms, such as microbial metabolites, intestinal permeability, and immune–metabolic signaling.^[32,33]^

Importantly, the magnitude of the observed microbial differences in this study was modest, and most associations did not survive the correction for multiple testing; therefore, these results should be interpreted cautiously. Nonetheless, the persistence of diagnostic group differences after adjustment for hs-CRP suggests that gut microbial alterations in AD may represent partially independent features of disease biology, rather than simple downstream correlates of the systemic inflammatory burden.

### 4.3. Associations between hs-CRP and gut microbial taxa

We also observed structured associations between hs-CRP levels and multiple gut microbial genera, with both positive and inverse relationships detected in regression and correlation analyses. Although only a subset of associations reached statistical significance after FDR correction, the consistency of directionality across analytic approaches supports a nonrandom relationship between systemic inflammatory tone, as reflected by hs-CRP, and gut microbial composition.

These findings are concordant with previous work showing that CRP reflects downstream hepatic responses to cytokine-mediated immune activation and may function as an integrative indicator of broader inflammatory signaling rather than a direct biological effector.^[34]^ In African populations, where baseline immune activation is shaped by lifelong exposure to infectious agents, diet, and environmental factors, the relationships between CRP and the gut microbiome may differ qualitatively from those observed in Western populations. Our results suggest that such contextual factors should be considered when interpreting inflammatory indicators in LMICs.

### 4.4. Microbiome modification of inflammation–cognition relationships

The most novel aspect of this study lies in exploratory interaction analyses, which suggest that gut microbial composition may modify the association between low-grade systemic inflammatory tone, as reflected by hs-CRP levels, and cognitive performance. Several genera demonstrated relatively larger CRP × taxon interaction coefficients than the main effects, indicating that the cognitive impact of inflammation differed depending on the microbial context. Although none of these interaction terms remained statistically significant after correction for multiple testing, their convergence in direction and magnitude across models supports the plausibility of microbiome-dependent modulation of inflammation–brain relationships.

These findings offer a potential explanation for the inconsistent associations reported between CRP levels and cognition across studies and populations. Rather than reflecting a direct or uniform effect of systemic inflammation on cognitive outcomes, CRP–cognition relationships may depend on broader biological contexts, including host–microbe interactions. Importantly, the taxa identified here should be viewed as markers within complex microbial networks, rather than isolated causal drivers.

### 4.5. Relevance to sub-Saharan Africa

This study contributes to a limited but expanding body of research on the biological correlates of cognitive aging in sub-Saharan Africa. Older adults in this region experience distinct life-course exposures, including recurrent infections, nutritional transitions, and constrained access to preventive healthcare, all of which may shape their long-term inflammatory trajectories and gut microbial composition. The modest and attenuated association between hs-CRP and cognition observed in this cohort, characterized by older age and restricted variability within the low-grade inflammation range, suggests that findings from high-income settings may not be directly generalizable to LMIC populations without careful consideration of the age structure and contextual exposures.

In this cross-sectional study of older Ugandans, hs-CRP was not independently associated with MoCA performance after adjusting for key demographic covariates. While gut microbiome profiling revealed nominal taxon-level variations across diagnostic and inflammatory profiles, no interactions survived the FDR correction. Given the modest sample size and exploratory nature of these analyses, hs-CRP should not be interpreted as a standalone marker of cognitive impairment in this setting. Larger prospective studies are needed to evaluate the longitudinal interplay between systemic inflammation, gut microbial communities, and cognitive trajectories in African cohorts.

## 5. Strengths

The key strengths of this study include the integration of cognitive assessment, systemic inflammation, and gut microbiome profiling in an understudied African population, as well as the use of complementary analytic approaches to assess the robustness of the observed associations. The explicit examination of interaction effects enhances the interpretability of inflammation–cognition relationships and addresses an important gap in the existing literature.

Although recruitment was community-based and conducted within Wakiso District, a geographically defined region, Wakiso is a highly cosmopolitan district that includes residents from diverse ethnic, cultural, and linguistic backgrounds, originating from multiple regions of Uganda. This diversity may partially enhance the representativeness of the sample; however, generalizability to other settings in Uganda and sub-Saharan Africa should still be interpreted with caution.

## 6. Limitations

This cross-sectional design precludes causal inference and limits the assessment of temporal relationships among inflammation, gut microbiome composition, and cognitive performance. The modest sample size constrained the statistical power, particularly for interaction analyses and correction for multiple testing, and may have limited the detection of subtle associations within a restricted range of low-grade systemic inflammation. CRP is a nonspecific marker that reflects peripheral inflammatory activity and may not capture neuroinflammatory processes occurring within the central nervous system. Finally, genus-level microbiome analyses lack functional resolution, underscoring the need for metagenomic and metabolomic approaches in future studies.

Although arcsine square-root transformation and prevalence filtering were applied to stabilize relative abundance data, the relative abundance remained inherently compositional. While our findings were corroborated by nonparametric analyses, future studies with larger cohorts should implement dedicated compositional toolsets (such as centered log-ratio transformations, ANCOM-BC, or MaAsLin2) to confirm these exploratory taxon-level signals.

Candidate genera showing nominal interaction signals, such as *Salmonella* and *Coxiella*, must be interpreted with caution. In short- or long-read 16S amplicon sequencing, low-abundance reads may represent bioinformatic misclassification within closely related families, low-level reagent/environmental background, or transient gut colonizers rather than genuine neuroinflammatory drivers.

## 7. Overall interpretation

In this study of older Ugandans, systemic inflammation, as measured by hs-CRP, showed at most a modest and nonindependent association with cognitive performance, whereas gut microbiome features demonstrated context-dependent relationships with inflammation and cognition. These findings should be interpreted cautiously given the cross-sectional design, limited sample size, and multiplicity of microbiome analyses, which increase the likelihood of chance findings despite correction for multiple testing. The largely null CRP–cognition associations are consistent with prior studies in older populations, in which inflammatory effects appear attenuated with advancing age, contrasting with stronger associations reported in midlife cohorts. Although several microbial taxa differed by diagnostic status and were associated with hs-CRP, the effect sizes were modest, and the findings were exploratory. Overall, the results suggest that CRP alone has limited utility as a marker of cognitive impairment in late life, but its relevance may depend on broader biological contexts, including host–microbiome interactions. Larger longitudinal studies are needed to clarify temporal relationships and evaluate integrated biomarker approaches in LMIC populations.

## 8. Implications and future directions

Taken together, these findings indicate that systemic inflammation, gut microbial composition, and cognitive performance are interrelated but not linearly linked in older Ugandans. While hs-CRP alone showed limited discriminatory value for cognitive performance in this late-life cohort, its relationship with cognition appears to depend on a broader biological context, including gut microbial composition. Longitudinal studies with larger sample sizes are needed to determine whether microbiome–inflammation interactions predict cognitive decline over time and whether integrated biomarker profiles can inform earlier risk identification.

Future research incorporating functional microbial data and additional inflammatory markers may further clarify the underlying mechanistic pathways and support the development of context-appropriate strategies for dementia risk assessment and prevention in LMICs.

## Acknowledgments

We thank Ms. Sylvia Nalwanga, Ms. Goretti Nambalirwa, and Ms. Nantamu Mariam for their invaluable support and assistance throughout the study.

## Author contributions

**Conceptualization:** Kamada Lwere.

**Data curation:** Kamada Lwere, Aisha Nazziwa, Ian Guyton Munabi.

**Formal analysis:** Kamada Lwere, Aisha Nazziwa, Ian Guyton Munabi.

**Funding acquisition:** Kamada Lwere, Mark Kaddumukasa.

**Investigation:** Kamada Lwere.

**Methodology:** Kamada Lwere.

**Project administration:** Kamada Lwere, Rheem Nakimbugwe.

**Resources:** Kamada Lwere.

**Software:** Kamada Lwere.

**Supervision:** Kamada Lwere, Noeline Nakasujja, Haruna Muwonge, Hakim Sendagire, Martha Sajatovic, Mark Kaddumukasa.

**Validation:** Kamada Lwere, Rheem Nakimbugwe.

**Visualization:** Kamada Lwere, Rita Nassanga.

**Writing – original draft:** Kamada Lwere.

**Writing – review & editing:** Kamada Lwere, Joy Louise Gumikiriza-Onoria, Denis Buwembo, William Buwembo, Samuel Kabwigu, James Kiguli Mukasa, Fred Kirya, Emmanuel Othieno, Rita Nassanga, Ian Guyton Munabi, Martha Sajatovic, Mark Kaddumukasa.
